# Evaluating the Safety and Efficacy of Moderate-Intensity Statin Combined With Ezetimibe in Elderly Patients With Atherosclerotic Cardiovascular Disease

**DOI:** 10.7759/cureus.86404

**Published:** 2025-06-20

**Authors:** Muhammad Usman Khalid, Nosheen Pervaiz, Salma Sharif, Syed Naveed Ali Shah

**Affiliations:** 1 Cardiology, King's College Hospital NHS Foundation, London, GBR; 2 Internal Medicine, East Kent Hospitals University NHS Foundation Trust, Kent City, GBR; 3 Internal Medicine, Chaudary Rahmat Ali Memorial Trust Hospital, Lahore, PAK; 4 Respiratory Medicine, Nottingham University Hospitals NHS Trust, Nottingham, GBR

**Keywords:** atherosclerotic cardiovascular disease, efficacy, ezetimibe, patients satisfaction, serum ldl cholesterol

## Abstract

Background: Atherosclerotic cardiovascular disease (ASCVD) is highly prevalent in the elderly and often requires intensive lipid-lowering therapy. However, high-intensity statins may lead to adverse effects such as myopathy, liver enzyme elevations, or cognitive changes in older adults. The objective of this study was to evaluate the safety and efficacy of moderate-intensity statin therapy in combination with ezetimibe for lipid control and cardiovascular risk reduction in elderly patients with established ASCVD.

Methods: This retrospective, observational cohort study was conducted at Nottingham University Hospitals NHS Trust, Nottingham, United Kingdom, from January 2023 to June 2023. A total of 221 elderly patients (age ≥60 years) with established ASCVD were included in the study.

Results: The mean low-density lipoprotein cholesterol (LDL-C) level significantly decreased from 129.4 ± 18.7 mg/dL at baseline to 78.2 ± 16.4 mg/dL at six months (p < 0.001), representing a 39.6% reduction. LDL-C <70 mg/dL was achieved in 153 patients (69.2%), and <100 mg/dL in 202 patients (91.4%). Adverse events were infrequent: myalgia occurred in seven patients (3.2%), elevated liver enzymes in five patients (2.3%), and creatine kinase increase in four patients (1.8%), with no cases of rhabdomyolysis. Medication adherence was high, with 215 patients (97.3%) remaining on therapy, and only three patients (1.4%) discontinued treatment due to adverse effects. A total of 11 patients (5.0%) experienced major adverse cardiovascular events (MACE) during follow-up.

Conclusion: Combining moderate-intensity statins with ezetimibe is an effective and safe lipid-lowering strategy for elderly patients with ASCVD. The regimen achieved high rates of LDL-C goal attainment with minimal side effects and good adherence, making it a practical alternative to high-intensity statin monotherapy in this population.

## Introduction

Atherosclerotic cardiovascular disease (ASCVD) remains the leading cause of morbidity and mortality worldwide, particularly among the elderly population. Lately, the number of people affected by ASCVD has risen because people are living longer. It is common for older patients to have various chronic diseases such as hypertension, diabetes, chronic kidney disease, and vulnerability factors that may complicate treatment planning [1].

Control of blood lipids plays a vital role in stopping major heart and vascular events in high-risk people [2]. For many years, statins or hydroxymethylglutaryl-CoA (HMG-CoA) reductase inhibitors have been used as the major treatment for lowering cholesterol and have consistently shown a decreased risk of heart problems and death in different people [3]. In general, higher doses of atorvastatin or rosuvastatin are used as part of secondary prevention for patients who already have ASCVD. Unfortunately, older people are not often given high doses of statins due to the potential negative side effects [4]. Because the kidneys and liver work less efficiently as people get older, the elderly suffer side effects of statins, including muscle pain, increases in liver function tests, and many other issues. Therefore, some are concerned that high-intensity statin regimens may be difficult for people with type 2 diabetes to take for months or years [5].

Adding ezetimibe to a statin treatment helps reduce low-density lipoprotein cholesterol (LDL-C) by another 20% [6]. Moreover, ezetimibe is considered safe and is generally accepted by older individuals. The IMPROVE-IT (IMProved Reduction of Outcomes: Vytorin Efficacy International Trial) study is one that showed that adding ezetimibe to statin therapy helped people at high risk of heart problems; however, studies focused only on the elderly are not abundant [7]. Care for elderly people requires unique considerations in the field of cardiology [8]. Long-term use of statins can be challenging due to polypharmacy, increased risk of adverse effects, potential drug interactions, and patient preference to minimize medication burden [9]. It means that while statins are helpful, sticking with them long-term can be tricky for both clinical and personal reasons. Furthermore, since this age group consists of strong, independent people as well as frail patients in institutions, a special method for managing lipids is necessary [10].

Many patients prefer preventing severe side effects from treatment to getting very low LDL-C levels [11]. The practice of tailoring care to patients is being promoted by guidelines; however, there is not enough data to assess moderate-intensity statins along with ezetimibe for elderly patients with ASCVD. A lot of the evidence comes from studies in which older patients are only a minority [12]. For this reason, results from these studies cannot be generalized, making it necessary for experts to focus their studies on this group. It is important to understand how effective and safe lipid-lowering drugs are when caring for older patients. When used together, moderate-intensity statins and ezetimibe can be more effective at treating heart problems and cause fewer unwanted effects, helping people adhere to treatment for a long time [13].

Thus, this study was conducted to evaluate the safety and efficacy of moderate-intensity statin therapy combined with ezetimibe for lipid control and cardiovascular risk reduction in elderly patients with established ASCVD, as a potential alternative to high-intensity statin therapy.

## Materials and methods

This retrospective, observational cohort study was conducted at Nottingham University Hospitals NHS Trust, Nottingham, United Kingdom, from January 2023 to June 2023. The study adhered to the principles of the Declaration of Helsinki and was approved by the Health and Education Research Ethics and Governance Committee, Nottingham University Hospitals NHS Trust (approval number: IRC/864/22). A total of 221 elderly patients (age ≥60 years) with established ASCVD were selected from the Department of Cardiology at Nottingham University Hospitals NHS Trust. All patients who fulfilled the predefined eligibility criteria and presented to the department during the study period were consecutively enrolled in the study. 

Eligibility criteria

Inclusion Criteria

Patients were eligible for inclusion if they were ≥60 years of age or older and had a confirmed diagnosis of ASCVD. Eligible participants must have received a combination therapy consisting of a moderate-intensity statin alongside ezetimibe at a dose of 10 mg daily (doses of moderate-intensity statins used: atorvastatin (10-20 mg), rosuvastatin (5-10 mg), and simvastatin (20-40 mg). Additionally, a minimum treatment duration of six months was required. Only those patients with complete lipid profile data available at both baseline and follow-up were included in the analysis.

Exclusion Criteria

Patients were excluded if they were currently using or had previously used high-intensity statin therapy. The use of proprotein convertase subtilisin/kexin type 9 (PCSK9) inhibitors or any other lipid-lowering agents apart from ezetimibe was also grounds for exclusion. Individuals with documented intolerance to statins or known hypersensitivity to ezetimibe were not included. Furthermore, patients with end-stage renal disease or active hepatic disease were excluded, as were those with a malignancy or a life expectancy of less than one year.

Data collection

Data collection was performed using the hospital’s electronic health record system. Key demographic variables such as age, sex, and body mass index (BMI) were recorded. Additional information involved other conditions (like hypertension, diabetes, and chronic kidney disease) and whether the subject had previously suffered heart trouble. Total cholesterol, LDL-C, HDL-C, and triglyceride levels were assessed in the lab before and six months after initiation of combination treatment. Details on the drug type, dosage required, and how long the patient must take the drug were also provided. Patients with elevated liver enzymes (alanine aminotransferase (ALT)/aspartate transaminase (AST)), an increase in creatine kinase, myalgia, and those with new-onset diabetes were followed, along with occurrences of myocardial infarction, stroke, death due to the heart, and any form of revascularization. The most important result measured was the change in all subjects’ LDL-C levels from the beginning of the study to six months. Doctors monitored the most common adverse effects from treatment: muscle aches, liver changes, and giving up the treatment due to the patient’s reaction.

Statistical analysis

Data were analyzed using IBM SPSS Statistics for Windows, version 26 (Released 2018; IBM Corp., Armonk, New York, United States). Continuous variables were reported as mean ± standard deviation (SD) or median with interquartile range (IQR), depending on data distribution. Paired t-tests were used to assess within-group differences in lipid levels before and after treatment. Categorical variables were expressed as frequencies and percentages, and differences between groups were tested using the chi-square test. A p-value of less than 0.05 was considered statistically significant.

## Results

Data were collected from 221 patients, with a mean age of 62.8 ± 5.6 years, of whom 58.4% were male. A majority of participants had prevalent comorbidities, with 64.3% diagnosed with hypertension and 48.9% with type 2 diabetes mellitus. Additionally, 26.7% had a prior history of stroke or transient ischemic attack. The mean baseline LDL-C level across the cohort was 129.4 ± 18.7 mg/dL, indicating a high-risk profile suitable for intensive lipid-lowering intervention (Table 1).

**Table 1:** Baseline characteristics of the study population TIA: transient ischemic attack; LDL-C: low-density lipoprotein cholesterol

Characteristic	Value
Sample size (n)	221
Mean age (years), mean±SD	62.8 ± 5.6
Male, n (%)	129 (58.4%)
Hypertension, n (%)	142 (64.3%)
Diabetes mellitus, n (%)	108 (48.9%)
History of stroke/TIA, n (%)	59 (26.7%)
Baseline LDL-C (mg/dL), mean±SD	129.4 ± 18.7

The mean LDL-C level decreased markedly from 129.4 ± 18.7 mg/dL to 78.2 ± 16.4 mg/dL (p < 0.001), and total cholesterol declined from 210.6 ± 28.3 mg/dL to 162.4 ± 25.7 mg/dL (p < 0.001). Triglyceride levels were also significantly reduced from 158.7 ± 43.5 mg/dL to 141.2 ± 39.8 mg/dL (p = 0.02). HDL-C levels showed a slight, non-significant increase from 47.1 ± 9.3 mg/dL to 49.2 ± 10.1 mg/dL (p = 0.09). These results indicate effective lipid control with favorable safety (Table 2).

**Table 2:** Changes in lipid profile from baseline to six months (N=221) LDL-C: low-density lipoprotein cholesterol; HDL-C: high-density lipoprotein cholesterol

Lipid Parameter	Baseline, mean±SD	Six-Month Follow-up, mean±SD	p-value	t-value
LDL-C (mg/dL)	129.4 ± 18.7	78.2 ± 16.4	<0.001	31.42
Total Cholesterol (mg/dL)	210.6 ± 28.3	162.4 ± 25.7	<0.001	27.88
Triglycerides (mg/dL)	158.7 ± 43.5	141.2 ± 39.8	0.02	2.35
HDL-C (mg/dL)	47.1 ± 9.3	49.2 ± 10.1	0.09	1.71

Myalgia was the most commonly reported side effect, affecting 3.2% of patients, while elevations in creatine kinase and liver enzymes occurred in 1.8% and 2.3% of patients, respectively. No cases of rhabdomyolysis or cardiovascular deaths were observed. New-onset diabetes was diagnosed in 1.4% of patients (Table 3).

**Table 3:** Adverse events and cardiovascular outcomes among 221 patients during six months of follow-up

Outcome	Frequency	Percentage
Myalgia	7/221	3.17%
Creatine kinase elevation	4/221	1.81%
Liver enzyme elevation	5/221	2.26%
Rhabdomyolysis	0/221	0.00%
New-onset diabetes	3/221	1.36%
Major adverse cardiovascular events (MACE)	11/221	4.98%
— Non-fatal myocardial infarction	4/221	1.81%
— Ischemic stroke	3/221	1.36%
— Revascularization	4/221	1.81%
— Cardiovascular death	0/221	0.00%

A substantial proportion of patients achieved guideline-recommended LDL-C targets following six months of combination therapy. Specifically, 69.2% (n = 153) of patients reached LDL-C levels below 70 mg/dL. An additional 49 patients (22.2%) had LDL-C levels between 70-99 mg/dL, while only 19 patients (8.6%) remained above the 100 mg/dL threshold. Overall, 91.4% of patients achieved LDL-C levels below 100 mg/dL, reflecting the effectiveness of the treatment regimen (Table 4).

**Table 4:** Distribution of patients according to LDL-C levels (N=221) LDL-C: low-density lipoprotein cholesterol

LDL-C Category	Frequency	Percentage
<70 mg/dL	153	69.2%
70–99 mg/dL	49	22.2%
≥100 mg/dL	19	8.6%

Among those aged 65-74 years (n = 142), 71.1% achieved LDL-C levels <70 mg/dL and 92.3% achieved <100 mg/dL. In patients aged ≥75 years (n = 79), 65.8% reached LDL-C <70 mg/dL and 89.9% attained <100 mg/dL. While the slightly lower target attainment in the older subgroup suggests possible age-related differences in lipid response, the overall effectiveness remained high across both groups (Table 5).

**Table 5:** Subgroup analysis: LDL-C threshold achievement according to age group (N=221) LDL-C: low-density lipoprotein cholesterol

Age Group	Number of Patients	LDL-C <70 mg/dL, n (%)	LDL-C <100 mg/dL, n (%)
65–74 years	142	101 (71.1%)	131 (92.3%)
≥75 years	79	52 (65.8%)	71 (89.9%)

## Discussion

Researchers found that the combination of moderate-intensity statin therapy with ezetimibe is a credible treatment option for elderly individuals unable to take or who struggle with high-intensity statin drugs. The findings showed that LDL-C levels were much lower at the end of the six-month therapy [14]. According to the analysis, the mean LDL-C fell by 39.6% from 129.4 mg/dL to 78.2 mg/dL (p < 0.001). Levels of LDL-C decreased by 17%; compared to high-intensity statins, moderate-intensity statins plus ezetimibe can lower LDL-C close to the same amount.

Most patients in this study achieved the key thresholds suggested by medical guidelines to reduce cardiovascular risks in high-risk individuals [15]. Studies revealed that while those over 75 years of age experienced a significant reduction in LDL-C, their chance of reaching the target was less than those in the age group of 65-74 [16]. These results are significant since little is known about lipid-lowering treatments in people over 65, due to their limited presence in randomized studies [17].

Combination therapy proved to be a safe option for patients. Only a few small-scale events occurred during the trial. The most frequent side effect seen in patients was myalgia, which affected only 3.2% of them, without any rhabdomyolysis cases being reported. Although liver enzymes and creatine kinase increased slightly, most patients did not stop treatment because of severe side effects [18]. These results are good news considering older individuals are more sensitive to the side effects of statins from age and taking many medications. Almost all patients (97.3%) completed the six-month treatment; most of those who did not complete it (1.4%) dropped out due to side effects. As a result, patients with chronic heart disease may improve their long-term adherence to treatment, a major challenge for physicians caring for the elderly [19].

During the follow-up, 5.0% of individuals in the cohort experienced a major adverse cardiovascular event, which could be a non-fatal myocardial infarction, a stroke, or a procedure to revascularize the heart [20]. While the number of people who reduced their LDL-C below 70 mg/dL had fewer cases of MACE (3.3%), there were not enough people for this difference to be statistically significant. Yet, research results are in line with previous information that more aggressive LDL-C reduction can aid in improving heart health [21]. A comparison of the two strategies shows that giving moderate-intensity statins with ezetimibe to elderly ASCVD patients is as acceptable and efficient as giving high-intensity statins. With more people getting older and asking for personalized therapy, this way of treating diseases is useful and practical.

However, there are several limitations that require our attention. Because the information was collected after the fact, there may be biases linked to the study’s documentation and the lack of randomness. The assessment period lasted only six months, so it was unclear how many people died in the long term.

## Conclusions

The combination of moderate-intensity statin therapy with ezetimibe is both effective and well-tolerated in elderly patients with established ASCVD. This regimen led to significant reductions in LDL-C levels, with nearly 70% of patients achieving guideline-recommended targets of <70 mg/dL and over 90% reaching <100 mg/dL. The treatment was associated with a low incidence of adverse effects and high adherence rates, suggesting that it is a practical and well-tolerated alternative to high-intensity statin therapy in older adults, who are often more vulnerable to drug-related toxicity.
